# Personalized wearable electrodermal sensing-based human skin hydration level detection for sports, health and wellbeing

**DOI:** 10.1038/s41598-022-07754-8

**Published:** 2022-03-08

**Authors:** Sidrah Liaqat, Kia Dashtipour, Ali Rizwan, Muhammad Usman, Syed Aziz Shah, Kamran Arshad, Khaled Assaleh, Naeem Ramzan

**Affiliations:** 1 School of Engineering and Computing, University of the West of Scotland, Paisely, PA1 2BE UK; 2 School of Computing, Edinburgh Napier University, Edinburgh, EH10 5DT Scotland UK; 3 Qatar Mobility Innovations Center, Qatar, Qatar Science & Technology Park, Doha, Qatar; 4 James Watt School of Engineering, University of Glasgow, Glasgow, UK; 5 Research Centre for Intelligent Healthcare, Coventry University, Coventry, UK; 6 Artificial Intelligence Research Centre College of Engineering and Information Technology, Ajman University, Ajman, UAE

**Keywords:** Biomedical engineering, Disease prevention

## Abstract

Personalized hydration level monitoring play vital role in sports, health, wellbeing and safety of a person while performing particular set of activities. Clinical staff must be mindful of numerous physiological symptoms that identify the optimum hydration specific to the person, event and environment. Hence, it becomes extremely critical to monitor the hydration levels in a human body to avoid potential complications and fatalities. Hydration tracking solutions available in the literature are either inefficient and invasive or require clinical trials. An efficient hydration monitoring system is very required, which can regularly track the hydration level, non-invasively. To this aim, this paper proposes a machine learning (ML) and deep learning (DL) enabled hydration tracking system, which can accurately estimate the hydration level in human skin using galvanic skin response (GSR) of human body. For this study, data is collected, in three different hydration states, namely hydrated, mild dehydration (8 hours of dehydration) and extreme mild dehydration (16 hours of dehydration), and three different body postures, such as sitting, standing and walking. Eight different ML algorithms and four different DL algorithms are trained on the collected GSR data. Their accuracies are compared and a hybrid (ML+DL) model is proposed to increase the estimation accuracy. It can be reported that hybrid Bi-LSTM algorithm can achieve an accuracy of 97.83%.

## Introduction

Water accounts for almost one half to two thirds of an average person’s body weight, where body fat can significantly influence the total body water. Human body fat tissues possesses less amount of water when compared to lean tissues where females generally have more fat in their body. Consequently, the amount of water in an average female (52 to 55%) is lower than an average male (60%). Due to the same reason, the percentage of body’s water in the elderly and obese is relatively lower than an average young human. Similarly, the percentage of body’s water during early childhood and at birth is higher (70%) than at young age.

Human body experiences dehydration occurs when quantify of water is lost due to external factors that include but are not limited to, illness, exertion with inadequate fluid intake, exposure to high temperature and use of diuretic medications. Dehydration creates a sodium imbalance in the body, which if not treated properly may result in mortality and morbidity. It is worth mentioning that in some individuals, the loss of as little as 2-3% of body fluid can cause physical and cognitive impairments.

Drinking enough amount of water is crucial for normal functioning of different parts of body. It is proven that prolonged and repeated cycles of dehydration may cause severe health complications including, but not limited to, heat injury, heatstroke, urinary tract infection, kidney problem, and even hypovolemic shocks^1^. Dehydration occurs when someone starts losing more liquids than she is taking in, which prevents many parts of the body to carry out normal functions. On the other hand, over-hydration is a condition, which may lead to water intoxication. This makes the salt and other electrolytes in the body too diluted to work properly. Over-hydration may cause edema and hyponatremia^1^. One of the most common causes of dehydration is diarrhoea, which is a prime cause of 300 annual deaths among the children living in the USA. The situation is even worse in the developing countries wherein around 2 million people die each year. According to World Health Organization (WHO)^2^, 4 billion cases of diarrhoea are registered worldwide annually.

To thin end, it becomes very important to regulate the hydration levels in a body to avoid diseases and complications. This can only be realised only through a hydration monitoring system^3^. The most common methods to measure the hydration levels are invasive in nature requiring isotopic dilution. For instance, Plasma Osmolality (PO) that measures the body’s electrolyte-water balance can only be checked through invasive means. Further, total body water (TBW) is another parameter to check the body hydration level, which is generally measured using invasive means. Although TBW can be estimated by measuring bio-electrical impedance (BIA) non-invasively, it is a complex method that cannot be used for continuous hydration level monitoring^4^. Further, it is an indirect measure of hydration level, which is estimated as a secondary parameter while measuring fat mass (FM) and fat-free mass (FFM) using BIA^3^. However, it is evident from BIA that there exists a correlation between hydration level and the electrical resistance of the skin, which generally varies from few ohms to thousands of ohms, depending on the hydration level of the body. Indeed, more than 99% of the body’s resistance against electrical flow comes from the skin^5^. Measuring the electrical resistance of the skin helps in the developing a non-invasive solution for monitoring hydration level in a body.Using electrodermal activity for hydration level monitoring is a biomarker of sympathetic nervous system activation and is considered one of the most sensitive and valid markers of emotional arousal^6^.

This paper presents a novel non-invasive skin-hydration detection technique exploiting the skin resistance of a body. In particular, electrodermal activity (EDA) sensor is used, which is specifically designed to measure the variations in the electrical properties of the skin^7^. The primary aim of EDA sensors is to study the sympathetic behaviour in humans using the electrical signals produced by the nervous system of the body. However, the sensor can be used to estimate the hydration level of the body. This is due to the reason that skin’s resistance significantly depends on the water level inside skin. In fact, skin’s resistance decreases with the water contents. In addition, the skin water level is directly correlated with the hydration level of the body^8^. Moreover, the hydration level is quantified in comparison with the change in the electrical dermal activity. Since, water is good conductor of electricity and when the human skin is hydrated, the EDA activity level will be higher. When the participant experiences dehydrated state, the EDA activity level will decrease as discussed through this work.

A limitation of using EDA sensor as an enabler of estimating hydration levels is the sensitivity of EDA towards stimuli of the nervous system, such as happiness, fear, humidity and temperature, etc. These internal and external stimuli cause rapid variations in the body resistance, where the variations due to hydration level does not exhibit impulsive behaviour. In order to resolve this issue, the data is collected for longer intervals of the time considering tonic activities instead of phasic. To this end, for the first time in the literature, a unique data collection is performed to accurately correlate the EDA sensors data with accurate measurements of hydration level in the body in extreme conditions, such continuous 16 hours of dehydration without any liquid intake. Indeed, the data is collected for three different states, namely, hydration, 8 hours of dehydration and 16 hours of dehydration. In addition, for each state, three different body postures are considered, sitting, standing and walking. It is worth mentioning that the data is collected for six female and ten male participants. The details of the data collection are mentioned in “Data description” section. An important contribution of this study is the auto-estimation of hydration level in the body using EDA data. In this regard, different feature sets and algorithms are investigated that gives the best performance. In particular, a hybrid model combining different deep learning (DL) and machine learning (ML) classifiers is designed to give the best estimation of hydration level in human skin and ultimately in the body. To summarise, this work presents a non-invasive system involving using off-the-shelf electrical dermal activity empowered by state-of-the-art deep learning algorithm. To the best of our knowledge, this is first study of monitoring hydration level of human skin under different conditions involving participants of various age range, weight, and in different posture.

The rest of the paper is organised as follows. “Related work” section summarises the state-of-the-art. The data collection details and description is presented in “Data description” section. The proposed methodology and the framework are presented in “Methodology” section. Simulation results are discussed in  “Results and Discussion and  Conclusion” sections concludes our work.

## Related work

Dehydration is an efficient predictor of morbidity and mortality in the patients^9,10^. The authors in^10^ assessed the complications of dehydration in the stroke patients after they are discharged from the hospital. It was found that dehydrated patients were likely to become more dependent on others than hydrated stroke patients. Similarly, over-hydration has been assessed in the literature for its link to many fatal diseases such as congestive heart failure and pulmonary edema^11–13^, confusion^14,15^, seizure, high blood pressure, and even death^16,17^.

Due to this, there is an increased interest in estimating hydration levels in a body in recent years. Indeed, an early detection of dehydration level is important to avoid serious complications. To this aim, it is desired to have a system for frequent identification of hydration levels. However, most of the methods proposed in the literature either rely on manual entry of water intake through mobile application or on some common signs and parameters, such as poor skin turgor, dry mucous membrane, urine colour, dry axilla, tachycardia, urine specific gravity, low systolic blood pressure, blood urea nitrogen to creatinine ratio, TBW, saliva flow rate, saliva osmolality, PO, and BIA^4,18–20^.

The limitations of some of the aforementioned methods is the requirement of clinical setting for the data collection. In addition, most of the methods are either invasive or require biochemical analysis of body liquids. Hence, it is very needed to have a wearable non-invasive hydration monitoring system that can timely identify the body’s hydration level with sufficient accuracy. Although BIA is a non-invasive method to estimate TBW, it is assumed as a complex solution requiring special equipment, which may not suitable for continuous monitoring. In addition, TBW is the by product of FM and FFM measurements in BIA. In order to fill the gap, some other non-invasive methods for measuring the body’s hydration level are proposed in the literature, using body temperature^21^, skin impedance^22^ and tracking the activity and water consumption of the user^23^.

Taking inspirations from the mentioned non-invasive techniques, we proposed a non-invasive method to estimate the hydration level relying on the galvanic skin response (GSR) or skin resistance level (SRL) of human body. Extending on the work presented in^24^, we further expand on the dataset, covering more states to include shorter and longer durations of fasting, *i.e.*, a fasting of 8 hours and 16 hours. Further, a body posture of walking is added. In addition, we propose a hybrid algorithm combining different ML and DL methods to give better accuracies on the identification of hydration level in the body.

**Figure 1:**
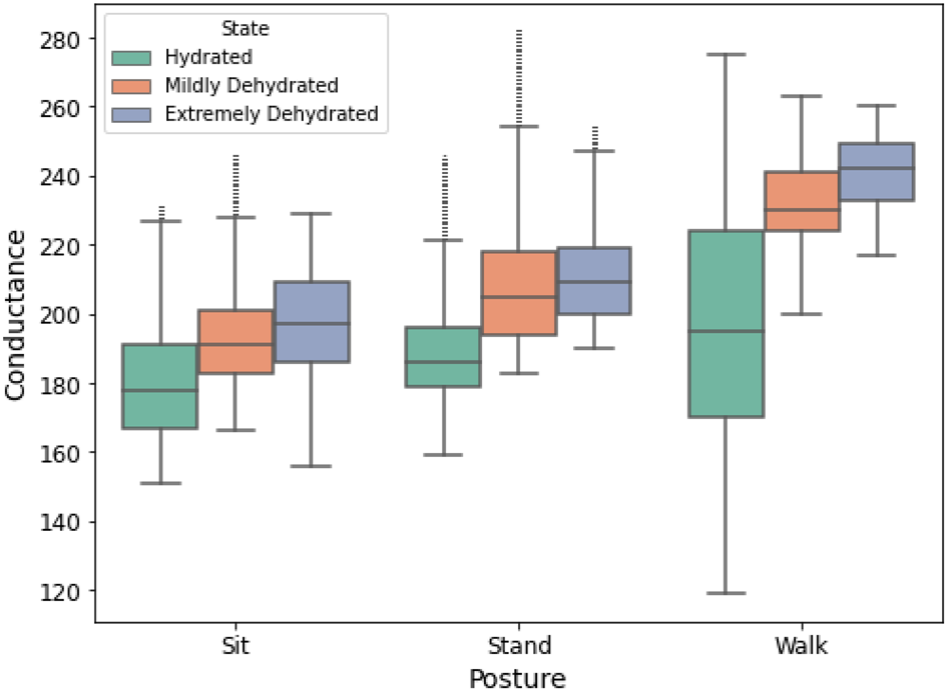
Skin conductance in three different states of hydration levels, ’Hydrated’, ’Mildly Dehdyrated’ and ’Extremely Dehdyrated’ and three body postures, sitting, standing and walking.

## Data description

Data used in this study is collected from 16 different participants after obtainting ethical approval from the University of the west of Scotland, including six females and ten males from different ethnicity groups. None of the participants has any known conditions of over hydration (edema or hyponatremia). Data was collected in three states labeled as ’Hydrated’, ’Mildly Dehdyrated’ and ’Extremely Dehdyrated’. For each state, the data was collected in three different body postures, *i.e.,* sitting, standing and walking. Distribution of data points for the raw data of skin conductance level in three different states and postures is shown in Fig. 1. It can be seen that the overall skin conductance increases with the dehydration level. Data was collected in three states labeled as ’Hydrated’, ’Mildly Dehdyrated’ and ’Extremely Dehdyrated’. For each state, the data was collected in three different body postures, *i.e.,* sitting, standing and walking. Distribution of data points for the raw data of skin conductance level in three different states and postures is shown in Fig.1. It can be seen that the overall skin conductance increases with the dehydration level. The data representing hydration state was collected after one hour of drinking two glasses of water at any time of the day and when the participants have been drinking water frequently. The data representing eight hours of dehydration was collected after waking up in the morning so that the participants had not drank water or any fluids for at least eight hours including sleep time. Whereas, the sixteen hours dehydration state represents the data collected during the month of Ramadan while the participants were fasting for a duration longer than sixteen hours. The data was collected two to three hours before breaking the fast.

A summary of the number of samples and duration of data samples collected is presented in Table 1. Data is collected in the format of five minutes samples each. Data is not collected for longer period in one go to avoid sweating on the palms caused by the continuous placement of electrodes for longer periods of time. Sweating can cause additional variation in the conductance level monitored via electrodes. From every participant, 2 samples of 5 minutes each were collected during all states. So, 30 minutes of data was collected during each state from each participant. In total, 288 samples with a cumulative length of 24 hours of data were collected from 16 participant with representative data of 90 minutes for each participant.

**Table 1:** Data samples statistical summary.

	Hydrated	Dehydrated 8 Hours	Dehydrated 16 Hours	Grand Total
Sampling Time (minutes)	5	5	5	
**For each participant**				
No. of Samples Sitting	2	2	2	6
No. of Samples Standing	2	2	2	6
No. of Samples Walking	2	2	2	6
Total duration (minutes)	30	30	30	90
No. of Particpants	16	16	16	16
Total Samples	96	96	96	288
Total duration 16 Particpant (minute)	480	480	480	1440

The data was collected using EDA sensor and BITalino toolkit after taking all necessary ethical approvals^25^. The data was collected at the highest possible resolution supported by BITalino, *i.e.,* a 16 bit resolution at a sampling frequency of 1MHz. In other words, 1000 samples are collected every second. The BITalino kit computes the GSR from the following formula.

1 $$\begin{aligned} GSR = \frac{1}{R}, \end{aligned}$$

where GSR is the skin conductance measured in $\mu S$ and *R* is the skin resistance in $M \Omega$, which can be represented as,

2 $$\begin{aligned} R = 1- \frac{C}{2^n}, \end{aligned}$$

where C is the digital representation of the value of the electrical signal collected by BITalino kit at resolution *n*.

## Methodology

**Figure 2:**
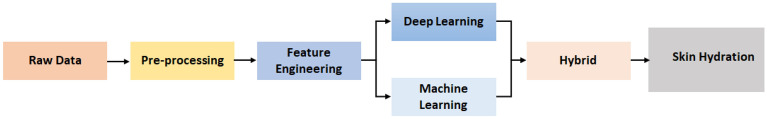
Overview of Proposed Framework.

The proposed framework is shown in Fig. 2. After data pre-processing and feature extraction, various different machine learning and deep learning algorithms are applied, including the hybrid approach to estimate the skin hydration.

### Window size selection

The data was initially collected for the intervals of 5 to 30 minutes and then spitted into smaller segments using a window operation. The window size, *W*, represents the size of each segment in seconds. Subsequently, feature extraction is performed on the segmented data. It is worth mentioning that different window sizes produce different data pattern after feature extraction. Considering this, an important task would be to identify the optimal window size that produces best results. To this aim, two different window sizes, $W \in {\{30,60}\}$, are selected and the best performing window is chosen among them. We decided not to use an overlapping window due to the reason that tonic features of GSR data are more relevant than the phasic features^26^.

### Feature extraction

In this study, we use the following nine statistical features: $F \in$ {Minimum, Mean, Standard Deviation, Percentile, Median, Kurtosi}, where *F* represents the feature space.

A feature space, *F*, of following nine statistical features is used: $F \in$ {Minimum, Mean, Standard Deviation, Percentile, Median, Kurtosi}. The values of each of the aforementioned features are calculated for the window sizes of 30 and 60 seconds. After feature extraction, it is important to determine the combination of features which generates the best performance for estimation of skin hydration. For that purpose, a genetic algorithms is applied to evaluate all combinations of the features for each algorithm. The data is segmented for each window size and above-mentioned nine features are extracted from each segment. For instance, when a window size of 30 seconds is selected, the data is segmented into non-overlapping segments of 30 seconds and features are extracted from each segment of 30 seconds data. This creates a vector of nice feature for each segment. Using these feature, the data-set is created.

### Implementation of ML/DL models

**Figure 3:**
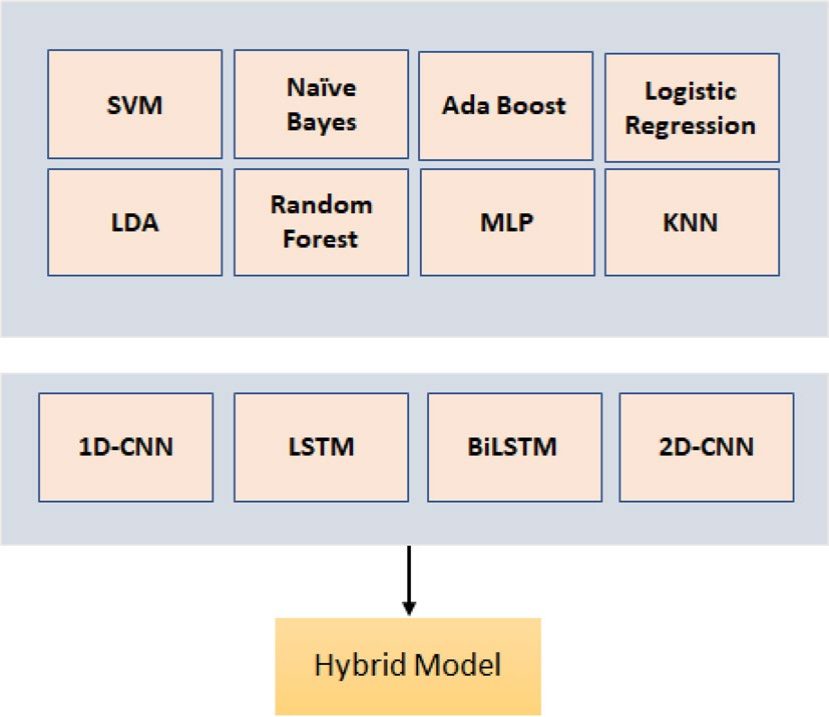
Proposed Hybrid Approach Framework.

In this work, we feed the predictions of ML and DL classifiers^27^ as an input to 1D-CNN, 2D-CNN, LSTM and BiLSTM architectures. The ML classifiers used in this study are support vector machine (SVM), k nearest neighbour (KNN), Naïve Bayes, multi-layer perception (MLP), random forest, logistic regression, linear discriminant analysis and Ada Boost. On the other hand, the DL classifiers used in this work are one dimensional convolutional neural networks (1D-CNN), 2D-CNN, long short-term memory (LSTM), and bi-direction LSTM (BiLSTM). The architecture of proposed hybrid model is shown in Fig. 3 where the parameters of each classifier are chosen after performing several experimental trials.

### Model evaluation

In order to evaluate the performance of the proposed approach, different evaluation metrics including accuracy, precision, recall and f-measure^27^ are used:

3 $$\begin{aligned} Precision =\frac{TP}{TP+FP}, \end{aligned}$$

where *P* represents the number of dehydrated instances, *N* represents the number of hydrated instances, *TP* (truly positive) represents the number of correctly detected dehydrated instances, and *FP* (false positive) represents the number of hydrated instances detected as dehydrated.

4 $$\begin{aligned} Recall =\frac{TP}{TP+FN}, \end{aligned}$$

where *FN* (false negative) represents the number of dehydrated instances detected as hydrated ones.

5 $$\begin{aligned} F\_measure= & {} 2*\frac{Precision*Recall}{Precision+Recall}, \end{aligned}$$

6 $$\begin{aligned} Accuracy (CCR)= & {} \frac{TP+TN}{TP+TN+FP+FN}, \end{aligned}$$

where *TN* (true negative) represents the number of correctly detected hydrated instances. The performance of the proposed algorithms is evaluated using correct classification rate (CCR), which a measure of overall classification accuracy (%). The performance is measured in all possible scenarios including window sizes, postures, states and the combination of extracted features. Once, the high performing algorithms are identified in all possible scenarios, the metrics like precision, recall and specificity are calculated to evaluate the detailed performance.

## Results and discussion

**Figure 4:**
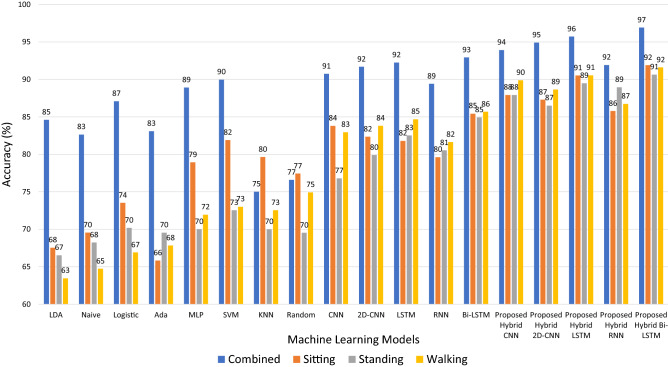
Proposed Hybrid Approach Framework.

In this section, we discuss about the results of the machine learning, deep learning and hybrid classification schemes used for the detection of three level of hydration. Figure 4 presents the performance results of eight models of classical machine learning, four models of deep learning with two version of LSTM, and five models developed using a hybrid scheme. The performance of the models is presented with CCR, the overall accuracy achieved in the detection of hydration, mild dehydration and extreme dehydration cases.

From Fig. 4, it can be seen clearly that the all models whether they be classical machine learning models or deep learning models they showed better accuracy on the combined data of all postures with few exceptions like KNN and Random forest where these two algorithms have shown similar behaviour of preforming better on the sitting data as compared to their performance on combined data where they showed their second best results. One potential reason for the over all better performance on the combined data, by the majority models, can be the availability of more data for training these models as compare to the data available to these models in posture specific scenarios where models are trained only on the separate data sets collected in sitting, standing or walking postures. More data for training means models have more information to learn the pattern in a particular state.

From Fig. 4, it can also be observed that the majority of the deep-learning models have preformed better as compared to the classical machine learning models. Even the RNN model with 89% accuracy, lower than that of other deep learning models, performs almost equally good as the best performing machine learning models e.g., SVM detecting hydration level with around90% accuracy. One of the main reason for such behaviour of better performance by the deep learning models can be the use of raw data instead of application on engineered features. For the classical machine learning models we have manually engineered the features form the raw data and model learn from the whatsoever information can be extracted from those features. Whereas in the case of deep learning models raw data is fed to the models so models have access to all data and they extract valuable information and correlation directly from the raw data which provides them an advantage over the classical machine learning models.

Another interesting trend evident from Fig. 4 is that the hybrid models perform even better then the other two families of models, the classical machine learning models and deep learning models with one exception of Hybrid RRN model. This comparatively low performance of the hybrid RNN model can be associated to the low performance of simple RNN model. The overall better performance of hybrid models can be credited to the fact that the hybrid model take advantage of the both, deep learning and classical machine learning, as they combine the positives of both types of models. They use deep learning models for the features extraction and machine learning models for the classification. It is a common finding that the deep learning models are good at extracting features whereas machine learning models are good at classification, so a combination of both models often leads to the improved performance. Considering overall detection accuracy as an evaluation criteria, it can be seen that the proposed hybrid Bi-LSTM model with an accuracy of around 97% outperforms majority of the other models and marginally preforms better than the other hybrid models like Hybrid LSTM and Hybrid 2D-CNN with an accuracy of around 96% and 95%, respectively. However, the overall accuracy alone may not represent a suitable evaluation metric. We have, therefore, used advanced evaluation metrics like precision, recall and *F*1 score to further evaluate the performance of the three models with highest overall accuracy. The Table 2 represents the performance of the best performing model hybrid Bi-LSTM on these advance metrics. From Fig. 4 and Table 2, it can be seen that hybrid Bi-LSTM model not only have the best overall accuracy but its detection rate for the each class like hydration, mildly dehydrated, and extremely dehydrated is also very high reflected from the corresponding precision recall and F1 score values in the Table 2. It can also be seen that the hybrid Bi-LSTM model has better precision, recall, F1-score on the combined dataset as compared to their values on the separate datasets of sitting, standing, and walking.

**Table 2:** Precision, Recall and F1-Score for three hydration states.

		Precision	Recall	F1-Score
Combined	Hydrated	0.96	0.96	0.96
	Mildly dehydrated	0.95	0.96	0.96
	Extremely Dehydrated	0.98	0.97	0.97
Sitting	Hydrated	0.89	0.91	0.90
	Mildly dehydrated	0.95	0.91	0.93
	Extremely Dehydrated	0.89	0.91	0.90
Standing	Hydrated	0.92	0.90	0.91
	Mildly dehydrated	0.88	0.90	0.89
	Extremely Dehydrated	0.91	0.90	0.90
Walking	Hydrated	0.89	0.91	0.90
	Mildly dehydrated	0.91	0.91	0.91
	Extremely Dehydrated	0.93	0.91	0.92

**Figure 5:**
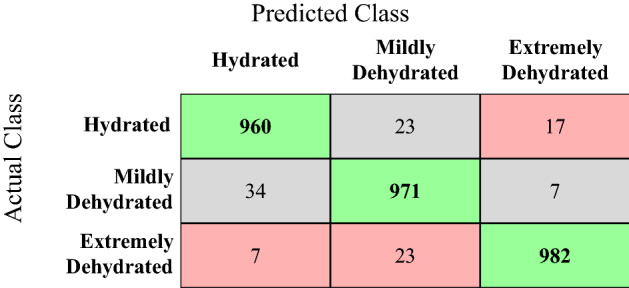
Actual level of hydration VS predicted level of hydration by the BiLSTM.

On the combined dataset the hybrid Bi-LSTM model yielded positive detection rate, also called recall value, of 0.96, 0.96, 0.97 for the detected hydrated state, mildly dehydrated state, and extremely dehydrated state, respectively. It means that this model could detect the 96% of the hydration cases, 96% of the mildly dehydration cases and 97% of the extremely dehydration cases correctly. That good performance is also reflected from the confusion matrix in Fig. 5, there are few cases of hydrated, mildly dehydrated and extremely dehydrated states, which are confused with each other. For example, from 1000 cases of hydrated state 960 are identified correctly by the model and 23 are wrongly identified as the cases of mild dehydration and 17 are miss-classified as the cases of extreme dehydration. Similarly, 34 cases of mild dehydration are miss-classified as the cases of hydrated state by the hybrid BiLSTM and only 7 are miss-classified as the cases of extreme dehydration. Overall hybrid BiLSTM has performed better in the identification of the extremely dehydrated cases as compared to the detection of mildly dehydrated and hydrated cases. Since correct detection of the cases of extreme dehydration is of the utmost importance here, so this behaviour of the proposed hybrid BiLSTM is very promising in this regard.

It is worth mentioning that the proposed best performing Hybrid BiLSTM also outperforms the state-of-the-art, as shown in the Table 3. It is also important to highlight that the best performing proposed model not only outperforms the state of the art in terms of accuracy and other metrics like precision, recall and F1 Score as listed in the Table 3, but it also has some key advantages over other state-of-the-art models. For example, models proposed in two very recent and relevant studies^3,24^ are not only inferior in terms of performance but they also have other limitations like capacity to identify only two level of hydration, hydrated or dehydrated. In addition, those models are trained on significantly lesser data, making them less generalized as compared to the hybrid model proposed in this work. With these findings it is concluded that the it is the first of its kind study in which a model is proposed for the detection of three level of hydration using non-invasive EDA sensor. The model proposed is not only unique in terms of its outcome, the number of hydration levels predicted, but it also outperforms the potential relevant stat-of-the-art.

**Table 3:** Comparison with State-of-the-art Approaches.

Ref	Accuracy	Precision	Recall	F-measure
Liaqat et al.^24^	91.53	0.91	0.90	0.91
Rizwan et al.^3^	85.63	0.85	0.84	0.85
Singth et al.^28^	70	0.72	0.71	0.72
Carrieri et al.^29^	73.91	0.73	0.72	0.73
Kulkarni et al.^30^	75.96	0.75	0.74	0.75
Our proposed approach	97.83	0.97	0.96	0.97

## Conclusion

In this study we have proposed a novel non-invasive method for the detection three levels of skin hydration in human-beings. We have collected data from 16 individuals in three states of hydration labeled as hydrated, mildly dehydrated and extremely dehydrated. Then we have exploited machine learning and deep learning algorithms and developed a hybrid model which detects the three level of hydration with an accuracy of around 97%. The model proposed is not only distinct in terms of the number of hydration levels predicted but it also outperforms potential relevant state-of-the-art for detecting skin hydration levels.

## References

[1] El-Sharkawy AM, Sahota O, Lobo DN (2015). Acute and chronic effects of hydration status on health. Nutr. Rev..

[2] Shah, S. A., Abbas, H., Imran, M. A. & Abbasi, Q. H. Rf sensing for healthcare applications. *Backscattering and RF Sensing for Future Wireless Communication* (2021).

[3] Rizwan A (2020). Non-invasive hydration level estimation in human body using galvanic skin response. IEEE Sensors J..

[4] Garrett DC (2017). Engineering approaches to assessing hydration status. IEEE Rev. Biomed. Eng..

[5] Fish, R. M. & Geddes, L. A. Conduction of electrical current to and through the human body: a review. *Eplasty* **9** (2009). PMC2763825 19907637

[6] Liu C (2016). Skin mechanical properties and hydration measured with mobile phone camera. IEEE Sensors J..

[7] Braithwaite JJ, Watson DG, Jones R, Rowe M (2013). A guide for analysing electrodermal activity (eda) & skin conductance responses (scrs) for psychological experiments. Psychophysiology.

[8] Huang X (2013). Epidermal impedance sensing sheets for precision hydration assessment and spatial mapping. IEEE Trans. Biomed. Eng..

[9] El-Sharkawy AM (2015). Hydration and outcome in older patients admitted to hospital (the hoop prospective cohort study). Age Ageing.

[10] Rowat A, Graham C, Dennis M (2012). Dehydration in hospital-admitted stroke patients: detection, frequency, and association. Stroke.

[11] Lobo DN (2004). Fluid, electrolytes and nutrition: physiological and clinical aspects. Proc. Nutr. Soc..

[12] Laine GA, Allen SJ, Katz J, Gabel JC, Drake RE (1986). Effect of systemic venous pressure elevation on lymph flow and lung edema formation. J. Appl. Physiol..

[13] Yoneda K (1980). Anatomic pathway of fluid leakage in fluid-overload pulmonary edema in mice. Am. J. Pathol..

[14] Veiga D (2012). Postoperative delirium in intensive care patients: risk factors and outcome. Braz. J. Anesthesiol..

[15] Prowle JR, Echeverri JE, Ligabo EV, Ronco C, Bellomo R (2010). Fluid balance and acute kidney injury. Nat. Rev. Nephrol..

[16] Wizemann V (2009). The mortality risk of overhydration in haemodialysis patients. Nephrol. Dial. Transplant..

[17] Boyd JH, Forbes J, Nakada T-A, Walley KR, Russell JA (2011). Fluid resuscitation in septic shock: a positive fluid balance and elevated central venous pressure are associated with increased mortality. Critical Care Med..

[18] Fortes MB (2015). Is this elderly patient dehydrated? diagnostic accuracy of hydration assessment using physical signs, urine, and saliva markers. J. Am. Med. Dir. Assoc..

[19] Armstrong LE (2005). Hydration assessment techniques. Nutr. Rev..

[20] Armstrong LE (2007). Assessing hydration status: the elusive gold standard. J. Am. College Nutr..

[21] Marsh, L. T. L. Hydration monitor. *U.S. Patent* (2013).

[22] Myers, A., Muth, J., Zhu, Y., Yao, S., Malhotra, A. Personal hydration monitor. *U.S. Patent* (2016).

[23] Gerald Sweeney, J. W. P., Cory McCluskey. Valve and cap system for a beverage container. *U.S. Patent* (2014).

[24] Liaqat S, Dashtipour K, Arshad K, Ramzan N (2020). Non invasive skin hydration level detection using machine learning. Electronics.

[25] Da Silva, H. P., Guerreiro, J., Lourenço, A., Fred, A. L. & Martins, R. Bitalino: A novel hardware framework for physiological computing. In *PhyCS*, 246–253 (2014).

[26] Society for Psychophysiological Research Ad Hoc Committee on Electrodermal Measures *et al.* Publication recommendations for electrodermal measurements. *Psychophysiology* **49**, 1017–1034 (2012).

[27] Taylor W (2020). A review of the state of the art in non-contact sensing for covid-19. Sensors.

[28] Singh, R., Shah, P. & Bagade, J. Skin texture analysis using machine learning. In *2016 Conference on Advances in Signal Processing (CASP)*, 494–497 (IEEE, 2016).

[29] Carrieri, A. P. *et al.* Explainable ai reveals key changes in skin microbiome associated with menopause, smoking, aging and skin hydration. *bioRxiv* (2020).

[30] Kulkarni, N., Compton, C., Luna, J. & Alam, M. A. U. A non-invasive context-aware dehydration alert system. In *Proceedings of the 22nd International Workshop on Mobile Computing Systems and Applications*, 157–159 (2021).

